# Vasohibin inhibits angiogenic sprouting *in vitro *and supports vascular maturation processes *in vivo*

**DOI:** 10.1186/1471-2407-9-284

**Published:** 2009-08-17

**Authors:** Johann Kern, Michael Steurer, Günther Gastl, Eberhard Gunsilius, Gerold Untergasser

**Affiliations:** 1 Division of Internal Medicine V, Tumor Biology & Angiogenesis Laboratory, Medical University Innsbruck, Innrain 66, A-6020 Innsbruck, Austria; 2 Division of Internal Medicine V, Laboratory for Molecular Genetics, Medical University Innsbruck, Anichstrasse 35, A-6020 Innsbruck, Austria

## Abstract

**Background:**

The murine homologue of human vasohibin (mVASH1), a putative antiangiogenic protein, was investigated for its effects on *in vitro *and *in vivo *angiogenesis.

**Methods:**

Cell growth and migration were analyzed in murine fibroblasts, smooth muscle cells and endothelial cells. Angiogenic sprouting was studied in human umbilical vein endothelial cells (HUVECs) in the spheroid sprouting assay. *In vivo *effects on blood vessel formation were investigated in the chorioallantoic membrane (CAM) assay and in the C57BL/6 melanoma xenograft model.

**Results:**

Purified murine and human VASH1 protein induced apoptosis of murine fibroblasts *in vitro*, but not of vascular aortic smooth muscle cells (AoSMC) or endothelial cells. Adenoviral overexpression of murine and human VASH1 inhibited capillary sprouting of HUVECs in the spheroid assay. Administration of recombinant murine and human VASH1 inhibited growth of large vessels in the CAM assay and promoted the formation of a dense, fine vascular network. Murine VASH1-overexpressing B16F10 melanomas displayed a reduction in large vessels and vascular area. Moreover, tumors showed more microvessels that stained positive for the mural cell markers α-smooth muscle cell actin (ASMA) and proteoglycan (NG2).

**Conclusion:**

Our data imply that murine VASH1 causes angiogenic remodelling by inhibiting angiogenic sprouting and large vessel growth, thereby supporting the formation of a vascular bed consisting predominantly of mature microvessels.

## Background

The human vasohibin 1 protein (hVASH1) has been postulated as an important negative feedback inhibitor of VEGF signaling and is thus a novel candidate for antiangiogenic therapies [1,2]. Despite the high degree of conservation and homology (>90%) of the vertebrate *VASH1 *gene, mouse and chicken have a shorter 289 aa protein lacking the N-terminal 76 aa (HomoloGene # 8941; http://www.ncbi.nlm.nih.gov). *Vasohibin-1 *(*hVASH1*) transcription in human endothelial cells has been shown to be upregulated by vascular endothelial growth factor (VEGF) [2,3], one of the key players in angiogenic processes [4]. The hVASH1 protein harbors a putative nuclear localization sequence, but has no signal peptide sequence within its 76 aa N-terminus. Thus, transfected cells show a cytoplasmic and nuclear accumulation of the protein under standard culture conditions [5]. Moreover, a shorter variant lacking the C-terminus (hVASH1B) has been identified in human endothelial cells [6]. This hVASH1B isoform has been shown to be a potent antiangiogenic protein in *in vitro *and *in vivo *angiogenesis assays [5]. The *VASH1 *gene seems to be expressed in a variety of tissues and organs during embryogenesis of mouse and chicken [7]. In particular, high transcriptional activity was found in the developing brain, eye, ear and joints. Hitherto, function of the shorter murine VASH-1 protein has not been studied.

In this study we analyzed whether the lack of the N-terminal protein domain affects angiogenic/antiangiogenic function. Due to the lack of specific and sensitive antibodies directed against murine VASH1 we generated a FLAG-tagged protein for detection of the recombinant protein after adenoviral or transposon-based overexpression. The affinity-purified recombinant FLAG-tag protein was tested in the angiogenic sprouting assay and the chicken chorioallantoic membrane assay. Moreover, overexpression of mVASH1 was analyzed in endothelial cells and in the B16F10 mouse melanoma model to study inhibitory effects on tumor growth and vascularization.

## Methods

### Cell culture

The murine B16F10 melanoma cell line was purchased from ATCC (American Type Culture Collection). Cells were grown in RPMI 1640 supplemented with 10% fetal calf serum, 1 × 10^5 ^IU/L penicillin, 100 mg/L streptomycin, 2-mmol/L glutamine in the presence of 5% CO_2_. Mouse vascular aortic smooth muscle cells (mASMC) were purchased from Dominion Pharmakine and cultivated in DMEM (Gibco BRL) with 10% fetal calf serum. Mouse embryonic fibroblasts (mFB) obtained from C129 mice were cultivated in IMDM (Gibco BRL) containing 10% fetal calf serum. HUVECs were isolated and propagated as described elsewhere [8]. Human microvascular endothelial cells (HMECs) were isolated from foreskin tissue according to the protocol of the CD31 Microbead Kit (Miltenyi Biotech) after overnight digestion in dispase II followed by a 1 h digestion step in collagenase I (both Sigma Biochemicals). All endothelial cells were cultured in EGM-2 (Cambrex) on type I collagen (Sigma-Aldrich) coated flasks.

### Cloning and generation of mVASH1 expression constructs

The *mVASH1 *cDNA was cloned from a mouse brain library of C57BL/6 mice by specific primers to exon 1 (for: 5-GTCAAGCTTACTGCCAGTGGATGAG) and exon 7 (rev: 5-TTATCTAGACACCCGGATCTGGTAC) of the genomic reference sequence on chromosome 12 (NT 039551.7). The amplified cDNA was subcloned in the pCRScript vector (Stratagene), and the nucleotide sequence verified by double strand sequencing. The cloned *mVASH1 *cDNA corresponded to the clone NM_177354 of the NCBI Entrez Nucleotide Data base http://www.ncbi.nlm.nih.gov. Due to the lack of specific antibodies against murine VASH1 the cDNA was cloned in frame with a short FLAG-tag (p3xFLAG CMS-14 vector, Sigma Biochemicals). This tag allows sensitive and specific detection of fusion proteins.

### Western/Dot Blot analysis

Cells were harvested and lysed in a buffer containing 10 mM Tris-HCl, pH 7, 0.2% Triton X-100 and protease inhibitors (Complete Mini EDTA-free; Roche Applied Science). Total protein (20 μg) was separated on 4%–20% SDS-PAGE and transferred to an Immuno-Blot™ polyvinylidene difluoride (PVDF) membrane (Bio-Rad). After blocking the membrane in 3% skim milk powder/PBS, it was probed with primary antibodies directed against FLAG-tag (0.1 μg/mL, Sigma Biochemicals, mouse monoclonal), human vasohibin (1 μg/mL, R&D systems, goat polyclonal anti human VASH1), carbonic anhydrase IX (CA9, 2 μg/mL, Santa Cruz Biotechnology, goat polyclonal anti human CA9), vascular endothelial growth factors (1 μg/mL, R&D systems, goat polyclonal anti mouse VEGF) or tubulin alpha (0.1 μg/mL, Sigma Biochemicals, mouse monoclonal anti human tubulin) and then detected with a 1: 2,500 dilution of an HRP-conjugated rabbit anti-mouse IgG or a 1:2,500 dilution HRP-conjugated rabbit anti-goat IgG. After washing, a chemoluminescent substrate (Super Signal West dura extended, Pierce) was added to the membrane, which was then exposed to the ECL Hyperfilm (GE Healthcare; Amersham Biosciences).

For Dot Blot analysis 1 μg total protein was spotted on a nitrocellulose membrane (BioRad). Blocking and detection were performed as described for the Western Blot.

### Purification of recombinant protein

Recombinant Flag-tagged mVASH1 was purified from cytosolic extracts according to the manufacturer's instructions using liquid chromatography columns and anti-FLAG M2 agarose beads (all SIGMA Biochemicals). The purified protein was quantified with a standardized FLAG-BAP fusion protein (SIGMA Biochemicals) by Western Blot analysis on the Chemidoc XRS using Quantity One software (BioRad). Human VASH1 (VASH1A, 365 aa) was produced and purified as described elsewhere [5].

### Adenoviruses

Replication-defective adenoviruses were generated with the AdEasy adenoviral vector system (Stratagene) according to the manufacturer's instructions. In brief, the *mVASH1-FLAG *cDNA was subcloned in the pShuttle CMV GFP-1 vector. Recombinant adenoviral DNA was generated in BJ5183 bacteria cells by means of a double-recombination event between co-transformed adenoviral backbone plasmid vector, pAdEasy-1, and a shuttle vector carrying the gene of interest. For generation of replication-defective adenovirus recombinant DNA was transfected into HEK293 cells by Lipofectamin 2000 (Invitrogen), after which cytosolic extracts were prepared. The human VASH1- and GFP-overexpressing adenoviruses were generated as described elsewhere [5,9]. All viral titres were determined by qPCR for the gene coding for the encapsulation signal (for:5-cgacggatgtggcaaaagt, rev: 5-cctaaaaccgcgcgaaaa) and the respective viral plasmid DNA standards.

### Flow cytometry

Cells were incubated with 10 nM solutions of the purified proteins and apoptosis and cell viability determined after 48 h. In brief, cells were detached by means of trypsin and then stained with FITC-labelled Annexin-V and 7-AAD (Beckman Coulter). After washing, cells were analyzed in a Cytomics-FC-500 cytometer using Cytomics RXP software (Beckman Coulter).

### Cell migration assay: Scratch assay

Cells were seeded into 6-well cell culture plates. Once at confluence, cells were serum-starved in medium containing 0.5% FCS over night, and then scratch injury was applied using a disposable pipette tip or rubber cell scraper (1-mm width). After injury, the monolayer was gently washed with PBS, and the medium was replaced with medium containing 1% FCS and 10 nM purified proteins or control FLAG-tag purified extract. Cell migration from the edge of the injured monolayer was examined and photographed 6 hours after scratching. Migrated cells were counted in 10 randomly selected high-power fields (HPF) adjacent to the scratch injury and were expressed as cells/mm^2^.

### Spheroid sprouting assay

The assay was performed as described elsewhere [10] with following modifications. HUVEC spheroids where generated overnight in hanging-drop culture consisting of 400 cells in EBM-2 medium, 2% FCS and 20% methylcellulose (Sigma Biochemicals). Spheroids were embedded in collagen type I from rat tail (BD) and stimulated with 25 ng/mL bFGF (Immunotools) or VEGF_165 _(Sigma Biochemicals) in the presence or absence of 10 nM purified recombinant proteins. Adenoviral transfection was performed 48 h before embedding of the spheroids to allow robust expression of the gene of interest.

### BrDU-incorporation assay

Five thousand cells/well were seeded in triplicates into a 96 well plate (Nunc) in culture medium and were allowed to adhere overnight before the culture medium was changed. Thereafter, cells were transfected with the adenovirus with a multiplicity of infection (MOI) of 10 virus/cell. After 2 day cells were pulsed for 24 h with a 10 μM dilution of BrDU (GE Healthcare) in culture medium. Then cells were washed, fixed and incorporated BrDU detected by a specific ELISA (GE Healthcare) in a ELISA reader (EL808, BioTek).

### Chorioallantoic membrane assay

The chick chorioallantoic membrane (CAM) assay has been used as an established *in vivo *model for screening for pro- and antiangiogenic proteins and drugs [11]. In brief, fertilized white leghorn chicken eggs (SPF eggs, each group n = 6) were purchased from Charles River (Kiesslegg) and incubated in an egg incubator at 37°C and 70% humidity (Compact S84, Grumbach) for four days. Subsequently, a window was cut in each eggshell and the underlying membrane. Eggs were incubated again for 4 h with the windows sealed (Durapor™ tape). Then, a Thermanox™ Ring (Nunc) was placed on the CAM and a 10 mM Tris-Glycine solution (pH 7.4) containing the purified recombinant protein (total 30 ng) or control extracts was added. Eggs were sealed and incubated for a further period of three days. Then they were opened and the CAM with the Thermanox™ ring was analyzed and photographed under a stereomicroscope with connected digital camera and flexible cold light (Olympus SZ51, Olympus E410). Blood vessels were counted inside the ring area (20 mm^2^). The allantoic vascular plexus was subclassified in large vessels (diameter > 50 μm) and microvessels (diameter < 50 μm).

### RT-PCR and qPCR

RNA was purified by cell lysis and nucleic acid extraction using the RNeasy Kit (Qiagen). Extracted total RNA was digested with DNAse I and then transcribed to cDNA using oligo-dT and hexanucleotide random primers (dN6) and the AMV-Reverse Transcriptase (all Promega). For analysis in the quantitative PCR 20 ng of each cDNA was used, 5 μL Sybr-green Mix (Bio-Rad) and 10 pMol of each primer: vasohibin (mVASH1 for: 5'-cccataccaagtgtgcctac, rev: 5'-agcctctttggtcatttcct), actin beta (*ACTB *for: 5-aagagctatgagctgcctga; rev: 5-tacggatgtcaacgtcacac), vascular growth factor (*VEGF *for: 5-ttactgctgtacctccacc; rev: 5-acaggacggcttgaagatg), transforming growth factor alpha (*TGFA *for: 5-tggctgtcctcattatcacc, rev: 5-tgggatcttcagaccactgt), angiopoietin 2 (*ANGPT2 *for: 5-gagcaaaccaccttcagaga; rev: 5-atcttctcggtgttggatga) basic fibroblast growth factor (*bFGF *for: 5-tccaagcagaagagagagga; rev: 5-tcagtgccacataccaactg), platelet-derived groth factor beta (*PDGFB *for: 5-ttccttcctctctgcta; rev: 5-tgagctttccaactcgactc)and angiopoietin 1 (*ANGPT1 *for: 5-aaatgcgcttctatgctaac, rev: 5-cagctttctttgcagctttc)

Analysis was performed within 50 cycles in the Bio-Rad iCycler (Bio-Rad). Data were collected and analyzed with the iCycler software.

### Generation of VASH1-overexpressing B16F10 cells

The generated cDNA-FLAG fusion protein sequence of the p3xFLAG-CMV14 vector (Sigma Biochemicals) was subcloned in the pT2 neo CIMS vector [9]. Murine B16F10 cells were transfected with the pT2 neo CMV *mVASH1 *FLAG vector. Cotransfection was performed with the pCMV SB11 vector encoding the sleeping beauty (SB) transposase. Clones resistant to neomycin were selected and propagated in ClonaCell medium (Stem Cell Technologies Inc.). All used VASH1-overexpressing clones (VH#1, VH#2, VH#4), and control clones (co#10, co#11, co#12) were analyzed for *mVASH1 *gene and protein expression before *in vivo *use.

### Mouse melanoma model

Animal experiments were performed after review and approval by the Austrian Government and the local committee for animal studies and established standards for animal handling. Female C57BL/6 mice, 5–6 weeks old, were purchased from Harlan Germany. Mice were subcutaneously inoculated in the right flank with 1 × 10^6 ^B16F10 cells dissolved in PBS. Experiments were performed using three mice per cell clone (total 18 animals). After one week tumors were measured each day using a caliper. After 14 days animals were sacrificed, the tumor weight determined and cryosections prepared for immunofluorescence staining. Moreover, after homogenization of the tissue mRNA and protein were extracted from all tumors to verify mVASH1 gene/protein expression *in vivo*.

### Immunofluorescence staining of blood vessels

Microvessel density (MVD) has been shown to correlate with angiogenic activity and tumor progression [12]. Thus, cryosections of B16 melanoma were prepared after freezing the removed tumor tissue in Tissue-Tek (Sakura Finetech) using methylbuthanol and liquid nitrogen. Thereafter, cryosections of 5 μm were cut and fixed in acetone. Sections were blocked in 3% BSA/PBS and incubated with a 1 μg/mL rat anti-mouse CD31 (PECAM-1, BD Biosciences), mouse anti-smooth muscle cell actin (ASMA; SIGMA Biochemicals) or rabbit anti mouse proteoglycan NG2 (Chemicon-Millipore). Detection was performed with an Alexa-488-labelled goat anti-rat (Molecular Probes, 1 μg/mL), Texas Red-labeled goat anti-mouse (Molecular Probes, 2 μg/mL) or TRITC-labeled swine anti-rabbit antibody (Dako Cytomation, 4 μg/mL). Nuclei were counterstained with DAPI (100 ng/mL, Molecular Probes). Tumors were examined with the Axiovert 200 M fluorescence microscope and Axiovert software (both Zeiss). Microvessel density (MVD; diameter < 50 μm) was determined by counting ten randomly chosen high-power fields (HPF 200×) from each tumor. Vascular lumens were traced and luminal areas were analyzed with the NIH ImageJ software.

### Statistical analyses

Statistical analyses were performed with GraphPad Prism™ software for Windows. Student's t Test and the Mann-Whitney U Test were used to study differences between two groups. Quantitative PCR data were calculated according to the delta Ct method described by Pfaffl *et al*. [13]. In brief, cDNAs of all tumor samples were normalized by the internal house-keeper gene actin beta (ACTB) and all cDNAs diluted to the same concentrations. Relative gene expression for each proangiogenic factor was calculated by the delta Ct method and the use of the mean Ct value of all control tumors (n = 9, relative expression of 1 ± standard error of the mean).

## Results

### Murine VASH1 induces apoptosis in embryonic fibroblasts

**Figure 1:**
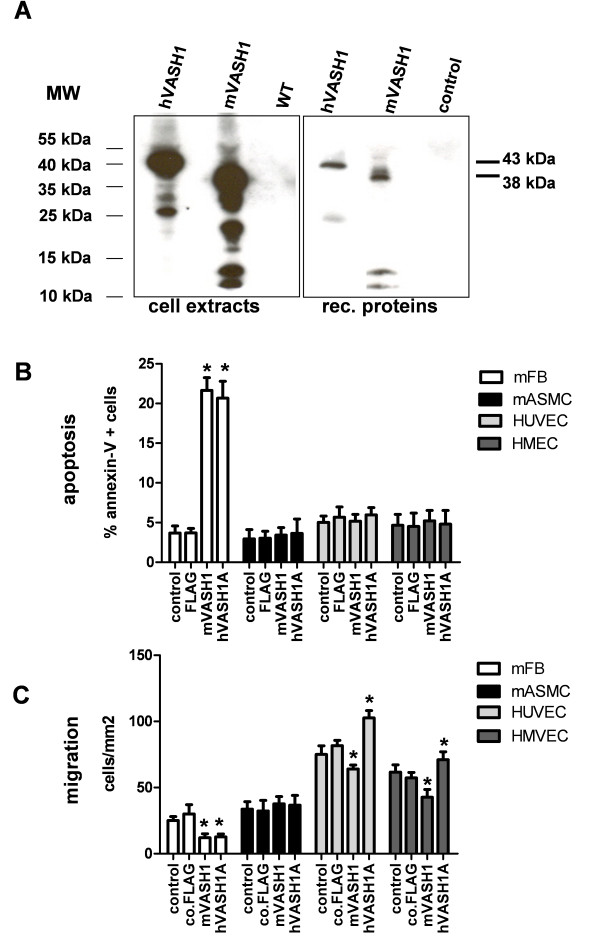
**Effects of recombinant vasohibins on cell viability of fibroblasts, smooth muscle and endothelial cells**. (A) Affinity purification of FLAG-tagged vasohibin proteins. Cell extracts of B16F10 cells with stable expression of human and murine VASH1 in comparison to wild-type cells (WT). Recombinant human and murine VASH1 proteins were analyzed by Western Blot after purification from cell extracts by affinity chromatography. Unspecific proteins of WT cells binding to the column are indicated as control. (B) Embryonic mouse fibroblasts (mFB), mouse aortic smooth muscle cells (mASMC), human umbilical vein (HUVEC) and microvascular endothelial cells (HMEC) were stimulated with 10 nM murine or human VASH1 protein or control extract (FLAG), after which apoptotic cells were counted by flow cytometry after Annexin-V/7AAD staining. Untreated cells were used as control. (C) Cell migration was analyzed in the scratch assay 6 h after stimulation with 10 nM mouse or human VASH1 or control protein. Stars indicate p values < 0.05.

Both human and mouse VASH1-FLAG protein were stably overexpressed in murine B16F10 cells for recombinant protein production (Figure 1a, left image). Flag-tagged protein was purified from whole cell extracts by affinity purification and quantified by Western Blot analysis with a monoclonal antibody specific for the C-terminal FLAG (Figure 1a, right image). Control FLAG extracts were generated by purifying extracts of the wild type B16F10 cells without Flag-tag fusion protein expression, i.e. traces of not specifically bound and eluted proteins. In addition to the full length protein, shorter proteolytic fragments were also purified.

Both VASH1 isoforms significantly increased cell apoptosis of mouse fibroblasts (Figure 1b, p < 0.05). Induction of apoptosis was observed only in murine fibroblasts (mFB) and not in mouse aortic smooth muscle cells (mASMC), human umbilical vein (HUVECs), or human microvascular endothelial cells (HMECs). Both, murine and human VASH1 significantly inhibited cell migration of mFB, but had no inhibitory effect on mASMC. Moreover, both vasohibins were tested on human endothelial cells were they showed an opposite effect on short-time cell migration (Figure 1c). Human VASH1 supported migration and murine VASH1 inhibited migration of human endothelial cells within 6 hours of incubation. Due to these effects on cell migration in the scratch assay, we tested both vasohibins in a more complex angiogenic sprouting assay based on three-dimensional cell-cell contacts and extracellular matrix degradation with proteases.

### Vasohibin inhibits angiogenic sprouting of endothelial cells *in vitro*

**Figure 2:**
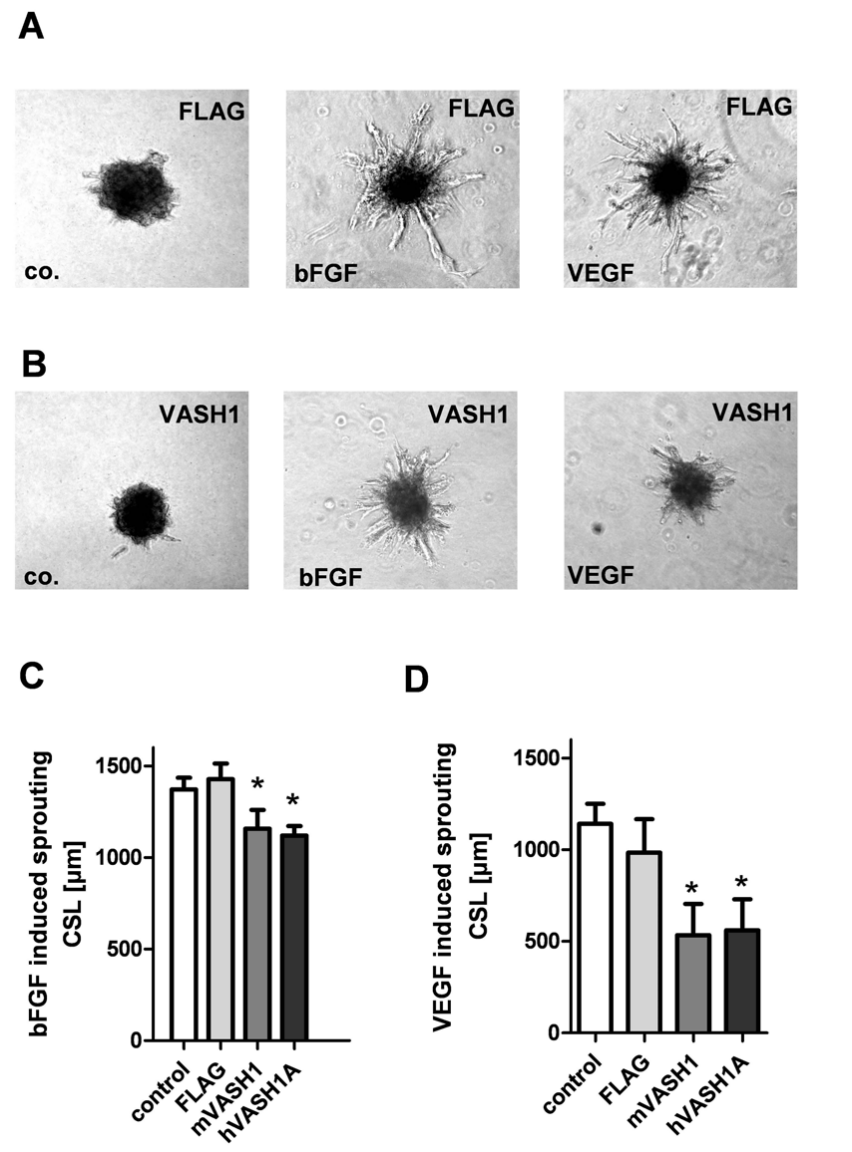
**Effects of recombinant vasohibins on angiogenic sprouting of endothelial cells**. (A) HUVEC spheroids were stimulated for 24 h with VEGF and bFGF in the presence of control FLAG protein to induce angiogenic sprouting into the collagen gel. (B) Angiogenic sprouting was inhibited by administering of 10 nM recombinant murine VASH1. (C) Effects of recombinant human and murine VASH1 (10 nM) on bFGF-induced angiogenic sprouting. (D) Effects of recombinant human and murine VASH1 (10 nM) on VEGF-induced angiogenic sprouting. FLAG indicates the control extract of the affinity purification. CSL indicates the cumulative sprout length of the spheroid and stars indicate p values < 0.05.

A three-dimensional angiogenic sprouting assay in rat collagen gel was performed to analyze the effect both vasohibin isoforms, human and mouse on VEGF- and bFGF-induced angiogenic sprouting. Both vascular growth factors induced strong angiogenic responses 24 h after stimulation when incubated with FLAG-control extract (Figure 2a). This was not observed after application of 10 nM murine or human VASH1 (Figure 2b). Application of recombinant human and murine VASH1 significantly inhibited bFGF-induced sprouting of spheroids (Figure 2c). The inhibitory effect was even more pronounced in VEGF-treated spheroids (Figure 2d).

**Figure 3:**
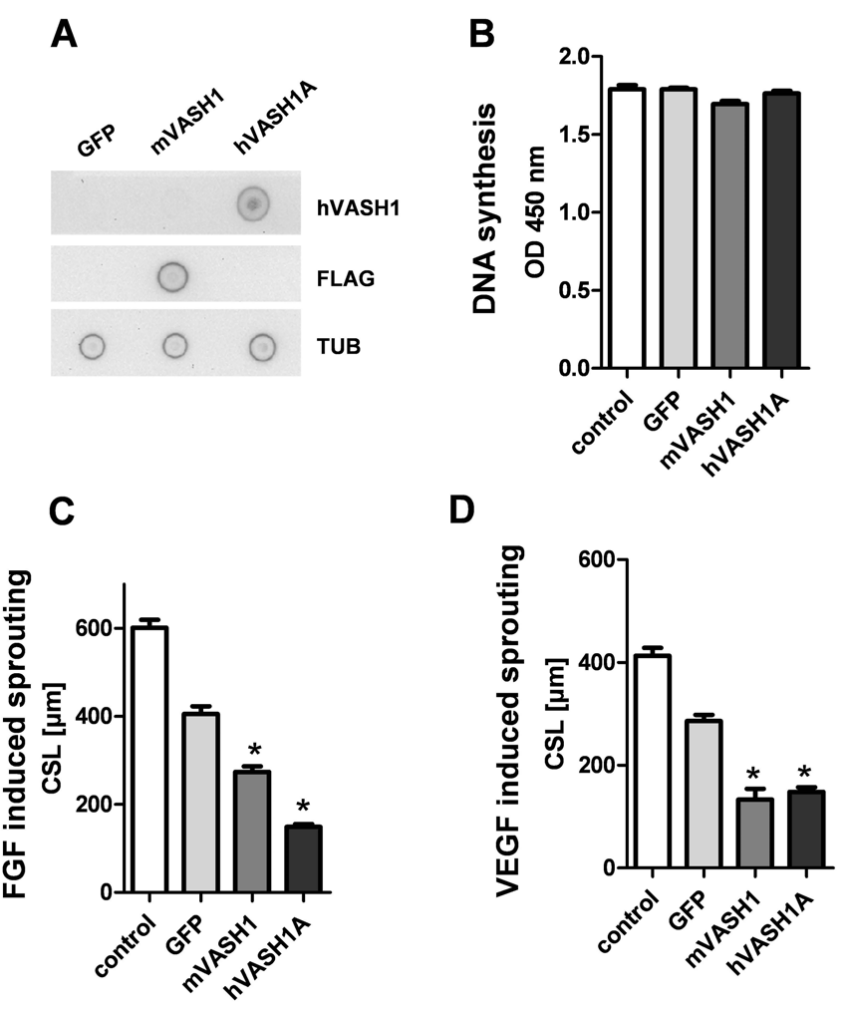
**Effects of adenoviral overexpression of *vasohibins *on cell proliferation and sprouting of endothelial cells**. (A) Dot Blot analysis of VASH1 protein expression 48 h after adenoviral transfection of HUVECs. The used MOI was 100 virus/cell. A monoclonal antibody directed against the FLAG-tag (FLAG) was used to detect FLAG proteins. Tubulin alpha (Tub) served as internal loading control. Note: the polyclonal antibody directed against human VASH1 (hVASH1) does not recognize murine VASH1. (B) Cell proliferation was analyzed after adenoviral overexpression of human and human vasohibins or GFP. BrDU incorporation was measured 48 h post transfection. (C) HUVEC spheroids were stimulated with bFGF to induce angiogenic sprouting two days after adenoviral transfection. CSL indicates cumulative sprout length of the spheroid. GFP was used as control for unspecific adenoviral effects. (D) Angiogenic sprouting induced by the growth factor VEGF was significantly inhibited by VASH1 overexpression. Stars indicate p values < 0.05.

The intracellular effects of VASH1 were tested after adenoviral overexpression of mouse and human VASH1. HUVECs showed strong expression of human and mouse VASH1 48 h after transfection (Figure 3a). Moreover, transfected cells were analyzed for inhibitory effects on cell proliferation. In comparison to GFP- transfected cells, both vasohibins did not significantly inhibit DNA synthesis in HUVECs (Figure 3b). Transfected cells were used to form spheroids and then stimulated with VEGF or bFGF for 24 h. In comparison to GFP-transfected cells, VASH1-transfected cells showed a reduction in bFGF- (Figure 3c) and VEGF- (Figure 3d) induced angiogenic sprouting.

### Vasohibin inhibits large-vessel growth and supports microvessel formation in the CAM assay

**Figure 4:**
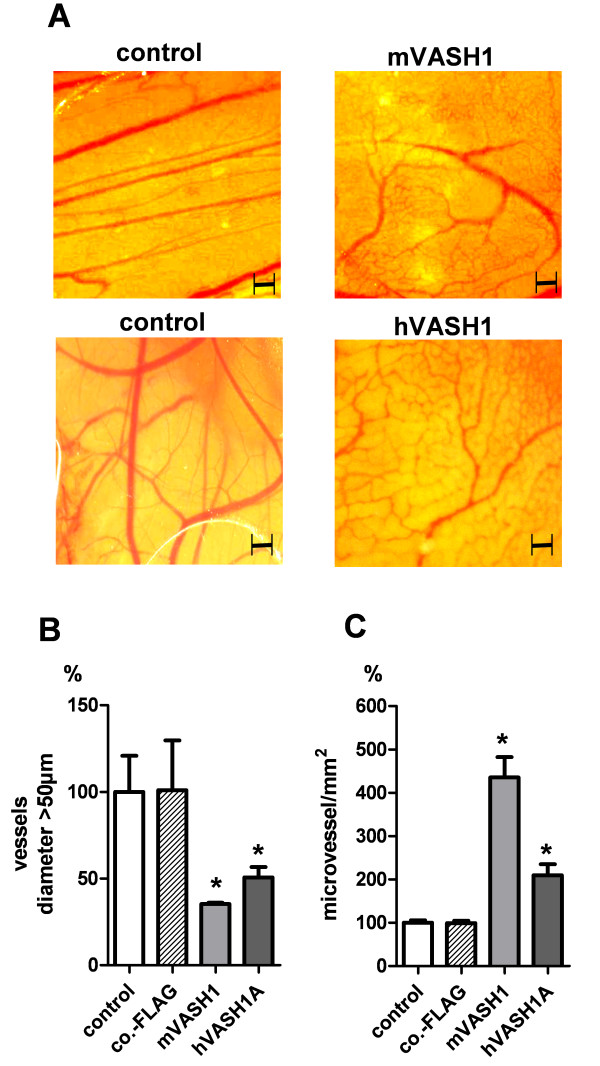
**Effects of recombinant VASH1 proteins on blood vessel formation in the CAM assay**. (A) Comparison of CAMs treated with 30 ng purified mVASH1 (n = 6), co-FLAG extract (n = 6) or 30 ng human VASH1 (n = 6). All blood vessels within the ring area (20 mm^2^) were counted. (B) Reduction of large vessels (diameter > 50 μm) in human and murine VASH1 treated CAMs. (C) Microvessels (diameter < 50 μm) were significantly increased in human and murine VASH1-treated CAMs. Stars indicate p values < 0.05. Controls indicate untreated CAMs (n = 6).

On the basis of our *in vitro *data in the HUVEC spheroid sprouting assay, we decided to test the murine vasohibin protein in direct comparison to the human isoform in the chicken chorioallantoic membrane (CAM) assay (Figure 4a). Application of 30 ng purified human VASH1/ring resulted in strong inhibition of large vessel formation after three days (p < 0.05, Figure 4b). Moreover, the number of visible blood-conducting microvessels significantly increased in human VASH1 treated CAMs (p < 0.05, Figure 4c). These effects were more pronounced after application of murine VASH1.

### Murine VASH1 supports microvessel maturation in the B16F10 melanoma model

**Figure 5:**
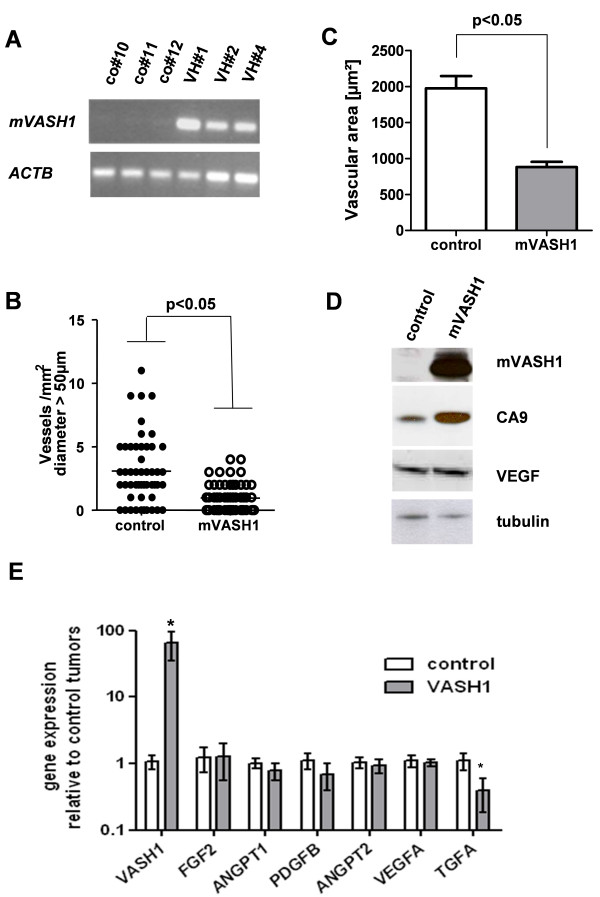
**Effects of stable overexpression of mVASH1 on tumor growth and large vessel growth**. B16F10 cell clones with mVASH1 overexpression (VH#1, 2, 4) or control clones (co#10, 11, 12) were subcutaneously transplanted in C57BL/6 mice and melanomas removed after 14 days. (A) *VASH1 *gene expression was analyzed in all tumor samples by RT-PCR. Actin beta (ACTB) served as housekeeping gene. (B) Statistical analysis of large vessels (diameter>50 μm) in controls and mVASH1-overexpressing tumors. (C) Analysis of vasular area in controls and mVASH1-overexpressing tumors (D) Western blot analysis of tissue protein extracts of a VASH1 overexpressing and control melanoma. Immunoreactive VASH1 protein was present in high amounts and also carbonic anhydrase 9 (CA9) was higher expressed in VASH1 overexpressing melanomas. VEGF protein was not altered. Tubulin alpha served as internal loading control. (E) Proangiogenic gene expression was analyzed by qPCR in controls and mVASH1-overexpressing tumors. Proangiogenic gene expression of VASH1 overexpressing tumors (n = 9) was quantified relative to the mean of all control tumors (n = 9). Stars indicate p values < 0.05 and bars mean ± SEM.

**Figure 6:**
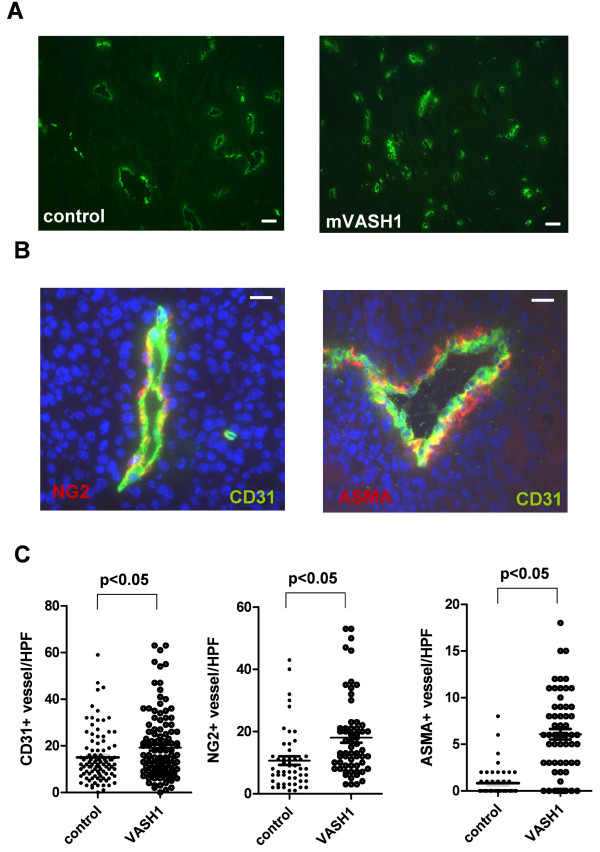
**Effects of stable overexpression of mVASH1 on tumor microvessel growth and maturation**. (A) Representative image of CD31 stained blood vessels in controls and VASH1 overexpressing melanomas. Bars indicate 50 μm. (B) Cryosections of all tumors were analyzed by co-staining for the endothelial marker CD31 together with the pericyte marker NG2 or the smooth muscle cell marker ASMA. Bars indicate 20 μm (C) Statistical analysis of microvessels in controls and mVASH1-overexpressing tumors. HPF indicates high power field (200×).

Murine B16F10 melanoma cells with stable overexpression of mVASH1 were generated. These cells were used to study the effects of mVASH1 on subcutaneous tumor growth and vascularization without any immunological interference due to overexpression of a xenoprotein. Interestingly, B16F10 cells themselves have weak endogenous *VASH1 *and strong *VEGF *and *ANGPT2 *gene expression (data not shown). *VASH1 *overexpression was retained in all three different cell clones (VH#1, VH#2, VH#4) clones analyzed within 14 days of *in vivo *growth (Figure 5a). Within the observed short time of two weeks, *mVASH1*-overexpressing melanomas did not show a significant decrease in tumor size (data not shown), but histologically large blood vessels with a diameter > 50 μm were nearly absent (Figure 5b, Figure 6a). Moreover, the vascular area in the tumors was strongly reduced (Figure 5c). Moreover, generated tumors showed immunoreactive Flag-tagged murine VASH1 protein (Figure 5d). Hypoxic regions in tumors were analyzed by the use of carbonic anhydrase 9 (CA9), one of the most sensitive endogenous sensors of HIF-1 activity. Analysis of tumor tissue revealed an increase of CA9 protein levels in mVASH1 overexpressing melanoma (figure 5d), whereas VEGF protein levels were not significantly altered.

Moreover. proangiogenic gene expression was analyzed in VASH1 overexpressing tumors (Figure 5e). Remarkably, mVASH1 overexpressing tumors showed a significant reduction of transforming growth factor -alpha (*TGFA*) gene expression, whereas *bFGF*, *VEGF*, *ANGPT1*, *ANGPT2 *and *PDGFB *gene expression differed not significantly. The downregulation of *TGFA *gene expression was also observed in all *in vitro *cultivated mVASH1 overexpressing cell clones (data not shown).

However, when microvessels in all tumors were analyzed with an antibody together with the mural cell markers ASMA or NG-2 (Figure 6b), mVASH1-overexpressing melanomas showed a significant increase in CD31+, NG2+/CD31+ and ASMA+/CD31+ microvessels (p < 0.05; Figure 6c).

## Discussion

*VASH1 *has been identified as a target gene of VEGF in human umbilical vein endothelial cells [2,3]. Moreover, the gene has been demonstrated to be strongly upregulated by VEGF in the mouse retinopathy model *in vivo *[14]. Despite the clear induction by VEGF, vasohibin has been shown to inhibit *in vitro *and *in vivo *angiogenesis after exogenous application and proteolytic modification [2,5]. Thus, it has been assumed that VASH1 functions as a negative feedback inhibitor of angiogenesis and might be a promising candidate structure for antiangiogenic therapies. The mechanism of action is still unclear, but vasohibin protein fragments might interfere in integrin/FAK signaling or inhibit angiogenic signaling pathways by interfering in ligand-receptor interactions. Hitherto, no receptor binding specifically VASH1 or its fragments has been identified. Additionally, vasohibin might be deposed in the extracellular matrix and thereby affect blood vessel formation and maturation [15] or be released into the blood circulation as shown for endostatin [16] and thereby inhibit angiogenic sprouting processes of endothelial cells.

This study compares the shorter murine VASH1 protein directly with the longer human homologue for antiangiogenic activities in a variety of *in vitro *and *in vivo *assays. Administration of recombinant mVASH1 neither inhibited proliferation nor induced apoptosis of endothelial cells *in vitro*. In line with our previous *in vitro *data with human VASH1A on HUVECs [5] the unprocessed human isoform either supported migration of human endothelial cells *in vitro*. This was not observed with the shorter murine VASH1, that slightly inhibited cell migration of human endothelial cells. Presumably, the N-terminal region modulates the function of human VASH1 and requires proteolytical removal to unmask an inhibitory domain responsible for inhibition of cell migration.

However, both VASH1 proteins induced apoptosis and inhibited cell migration in fibroblasts. Thus, VASH1 released from endothelial cells or cells of the immune system [5] could inhibit fibroblast proliferation in the tumor stroma and inhibit pro-angiogenic tissue remodeling in the reactive stroma of the tumor and support differentiation processes of mural cells.

Interestingly, exogenously applied recombinant protein as well as intracellular overexpression of the *mVASH1 *gene strongly inhibited angiogenic sprouting of HUVEC spheroids in the three-dimensional collagen gel. This observation is a novel finding and indicates that in order to act VASH1 needs to interact with a complex extracellular matrix, proteases and/or three-dimensional cell-cell contacts.

Exogenous administration of recombinant murine and human VASH1 in the CAM assay strongly induced a remodeling of the developing embryonic allantoic vascular plexus. In comparison to controls, large vessel formation was strongly inhibited and the number of blood-conducting microvessels was significantly increased. These *in vivo *data and the *in vitro *observation that VASH1 inhibited spheroid sprouting processes might indicate that VASH1 has additional functions. The protein might acts as a morphogen in vascular remodeling by inhibiting the formation of larger vessels in favor of a vascular bed consisting primarily of small capillaries. Whether the observed increases of microvessels are the consequence of collapsing large vessels or hypoxia in the CAM after VASH1 induced destabilization of larger vessels remains to be investigated in future studies.

Apart from its effects on physiological angiogenesis murine VASH1 was also studied in a mouse tumor model to analyze its effects on tumor growth and vascularization. This was done in the B16 melanoma model, a tumor model with a strong proangiogenic phenotype, i.e. production and secretion of VEGF and angiopoietin-2 [17].

In agreement with Watanabe et al. [2] we found no influence on *in vitro *proliferation of tumor cells but a significant reduction in large vessels and vascular area in *mVASH1*-overexpressing melanomas. Simultaneously, we observed an increase of microvessels in *mVASH1*-overexpressing tumors. This might be due to hypoxia after large vessel regression, since Hosaka et al. observed a positive correlation between VASH1 expression in blood vessels and HIF-1α expression in tumor cells of human non-small cell lung carcinoma [18]. At least in the final stages of our melanoma we found an increases of carbonic anhydrase protein levels, an enzyme induced by hypoxia and a sensor of HIF1α transcription factor activity [19]. However VEGF, an other important target of hypoxia was not increased in the final stages of mVASH1 overexpressing melanomas. These observations can be explained that either hypoxia did not always provoke VEGF upregulation in tumors [20] or that VEGF was upregulated in early stages and normalized at later stages of tumor progression.

Moreover, microvessels were more mature, i.e. they had more mural cell coverage as shown by costaining for pericytes (NG2) and smooth muscle cells (ASMA). These observation is in line with the recent observations in VASH1 knock-out mice, that show defects in blood vessel maturation of Lewis lung carcinoma xenografts [18,21].

Presumably, the fast-growing subcutaneous melanoma model prevented us from noticing a reduction in tumor size, as Watanabe et al. [2] observed in the Lewis lung carcinoma xenograft model overexpressing *the human VASH1A*. Therefore, the efficiency of the antiangiogenic effect of VASH1 might strongly depend on the tumor type analyzed. Additional tumor models have to be studied to identify tumor types that are sensitive to an antiangiogenic therapy consisting of gene therapy or systemically administered VASH1 protein or peptides.

## Conclusion

Although vasohibin has been proposed to be primarily an antiangiogenic protein, we could further demonstrate that it acts also as a morphogen in the vessel maturation process *in vivo*. Overexpression in tumor xenografts led to a reduction of larger tumor vessels and supported the formation of a dense microvessel network with more mature vessels covered by mural cells, such as pericytes and smooth muscle cells.

## Competing interests

The authors declare that they have no competing interests.

## Authors' contributions

GU conceived the study, abstracted data, drafted and revised the manuscript. JK carried out all angiogenic assays. MS performed the statistical analysis. EG and GG participated in discussion of results. The final version of the manuscript was read and approved by all authors.

## Pre-publication history

The pre-publication history for this paper can be accessed here:

http://www.biomedcentral.com/1471-2407/9/284/prepub
